# A novel electronic key-controlled expander for precise asymmetric palatal expansion

**DOI:** 10.3389/fdmed.2025.1735298

**Published:** 2026-01-12

**Authors:** Nora Alhazmi

**Affiliations:** 1 Department of Preventive Dental Sciences, College of Dentistry, King Saud bin Abdulaziz University for Health Sciences, Riyadh, Saudi Arabia; 2 King Abdullah International Medical Research Center, Riyadh, Saudi Arabia; 3 Ministry of The National Guard Health Affairs, Riyadh, Saudi Arabia

**Keywords:** asymmetric expansion, digital orthodontic appliance, electronic expander, orthodontics, palatal expansion

## Abstract

Achieving precise, comfortable, and asymmetric maxillary expansion remains a clinical challenge in orthodontics. Conventional expanders, such as the Hyrax, depend on manual activation by caregivers, often leading to inaccurate screw turns, patient discomfort, mucosal injury, and inconsistent results. Moreover, their mechanical design limits controlled asymmetric expansion, reducing effectiveness in unilateral crossbites. This brief research report introduces a proof-of-concept electronic, key-controlled palatal expander designed to enhance precision, safety, and ease of use. The concept integrates an electronic activation system with a multi-keyhole design, allowing both symmetrical and asymmetrical expansion tailored to individual patient needs. To assess the device's mechanical behavior, a functional digital simulation was conducted using the finite element method (ANSYS 2024 R1, ANSYS Inc., USA). Activation of the center screw produced smooth, stable, and symmetric bilateral expansion, with a 1 mm screw advancement generating approximately 1.08 mm of lateral displacement. Selective activation of lateral keyholes yielded illustrative unilateral movement, with each 1 mm screw activation resulting in approximately 1.9 mm of displacement, demonstrating the device's potential for controlled asymmetric expansion under the modeled conditions. Currently at the conceptual and design stage, the device has not undergone bench or clinical testing. However, the mechanical simulation supports the feasibility of a digitally guided expander capable of delivering controlled and customizable expansion in theory while reducing reliance on caregiver-performed activations. This innovation may offer a safer and more precise alternative to conventional devices, although all proposed advantages remain preliminary and require experimental and clinical validation before clinical use.

## 1 Introduction

Maxillary transverse deficiencies and posterior crossbites are among the primary predictors of dentofacial deformities, with a high prevalence in dental practice (1). A posterior crossbite can be unilateral or bilateral, and is a transverse arch discrepancy in which the palatal cusps of one or more maxillary teeth fail to occlude within the central fossa of the opposing mandibular arch (2). Its etiology may include premature loss or prolonged retention of deciduous teeth, genetic predisposition, palatal clefts, dental crowding, oral habits, temporomandibular joint dysfunction, or arch deficiencies (3). If left untreated, a posterior crossbite can lead to abnormal mandibular movements, temporomandibular joint disorders, and craniofacial asymmetry in children, adolescents, and adults (4).

The primary treatment for maxillary transverse deficiencies is rapid maxillary expansion, a technique introduced in the 1860s that facilitates both skeletal and dental expansion of the maxilla (5). The Hyrax expander is the most commonly used rapid maxillary expansion appliance (6). The Hyrax expander, which relies on a manual key for activation. However, the conventional design presents several challenges. Manual activation relies on caregivers and is associated with challenges related to precision and patient compliance. Additionally, improper use of the key can lead to palatal mucosal lacerations (7). Another major limitation of the Hyrax expander is its inability to perform asymmetric expansion (8), making it unsuitable for unilateral crossbites. In such cases, clinicians typically resort to symmetric expansion followed by constriction on the unaffected side, a process known as round tripping. Advancements in digitalization and modern technology have significantly affected orthodontics, offering promising improvements in treatment outcomes (9, 10).

In this study, we present an innovative design and mechanics of the electronic key-controlled expander. This device addresses several limitation of the traditional Hyrax expander and has the potential to allow for customizable expansion, reduced patient visits, and enhanced safety. To preliminarily evaluate its mechanical behavior, we also performed a finite element analysis (FEA) to simulate its activation patterns and expansion mechanics. Although these benefits remain to be validated clinically, this conceptual work provides the foundation for further development.

## 2 Materials and methods

### 2.1 Device design description

**Figure 1:**
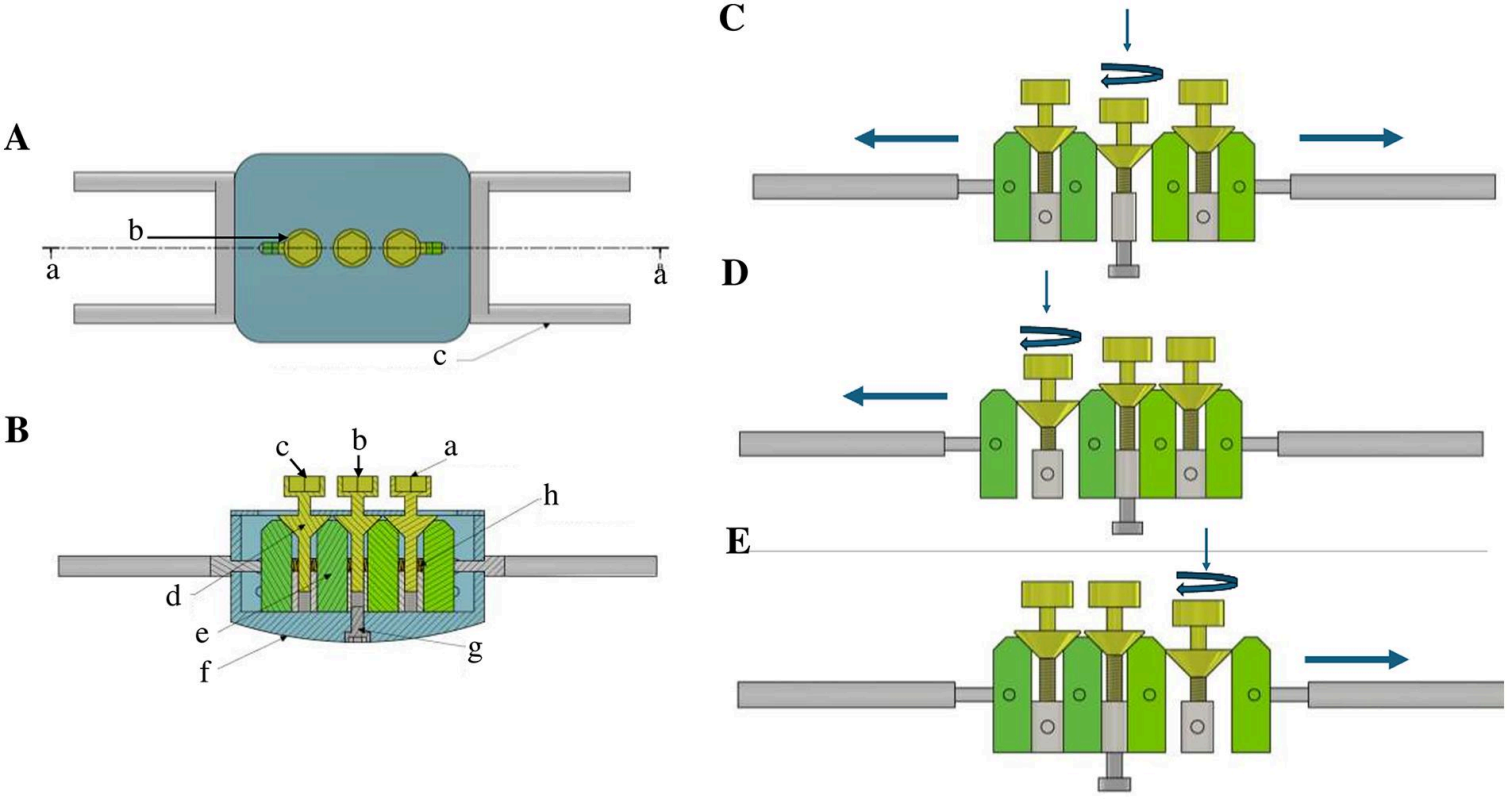
Structural design and mechanical behavior of the innovative expander. (A) Central vertical cross-section of the device showing: **(a)** section-plane reference; **(b)** keyhole; **(c)** metal arm transferring force to the jaw. (B) Internal view corresponding to panel A demonstrating: **(A–C)** keyholes; **(d)** screw wedge descending with clockwise rotation; **(e)** moving block; **(f)** rounded contours preventing food accumulation; **(g)** central screw block fixed from below to restrict movement to the lateral wedge blocks; **(h)** recoil springs. **(C)** Symmetric expansion mechanics: activation of the central keyhole producing approximately 0.25 mm separation per turn. **(D)** Left-sided asymmetric expansion mechanics: selective activation of the left keyhole generating primarily left-sided movement. **(E)** Right-sided asymmetric expansion mechanics: selective activation of the right keyhole generating primarily right-sided movement.

**Figure 2:**
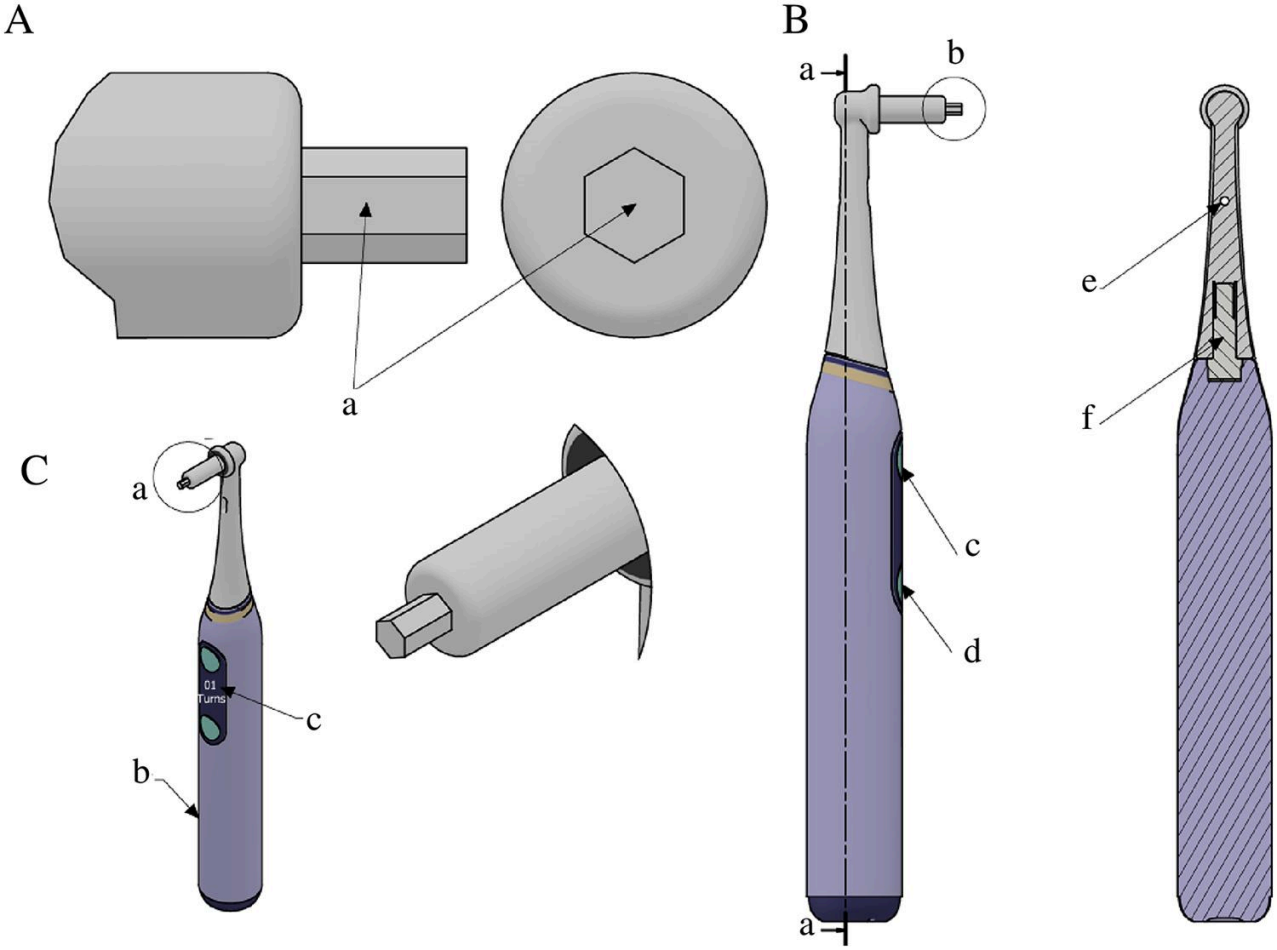
Electronic key components. **(A)** Head of the electronic key: **(a)** hexagonal shape matching the expander keyhole. **(B)** Sectional view: **(a)** dotted line indicating section plane; **(b)** electronic key head; **(c)** motor power button; **(d)** LED switch button; **(e)** LED light; **(f)** motor. **(C)** Auxiliary views: **(a)** electronic key head; **(b)** handle of the electronic key; **(c)** display screen showing the number of turns and direction of the activation movement.

The electronic key-controlled palatal expander is a novel orthodontic device designed to enhance treatment precision, safety, and patient comfort. The device consists of two components: the innovative expander attached to the patient's mouth and the electronic key control (Figures 1A–E, 2A–C). The expander resembles a traditional Hyrax in shape, with four metal arms attached to the molar bands and side arms to transfer forces to the jaw. Unlike the Hyrax, the inner palatal surface is convex and the expander contains three keyholes instead of one (Figure 1B and Supplementary Figure S1).

The inner structure of the innovative expander is illustrated in Figure 1B. The central screw block is fixed at the base, allowing only the side wedge blocks to move. Each keyhole contains a wedge, which descends with each turn, enabling block separation. Slots guide block movement, and springs provide recoil and stabilization (Supplementary Figure S2).

The device features an advanced electronic key mechanism that enables conceptual digitally guided asymmetric expansion, which may produce minor movement on the opposite side under modeled conditions, potentially mitigated with appropriate anchorage, and has not yet been clinically tested (Figures 2A–C). The electronic key features a hexagonal head that fits the keyhole and rotates clockwise or counterclockwise. The ergonomic handle includes a power button, a LED light to illuminate the keyhole, and a display screen showing the number of turns and rotation direction. Additionally, the system integrates with a mobile application, allowing orthodontists and patients to monitor activation in real time. This innovation has the potential to enable more accurate and consistent activation, reduces reliance on caregivers, and minimizes potential treatment errors.

### 2.2 Device design biomechanics

A key feature of this device is its ability to perform conceptual asymmetrical expansion via the three keyholes, tailored to individual patient needs, which may result in minor contralateral movement under modeled conditions. Figures 1C–E demonstrates both symmetrical and illustrative asymmetrical activation.

- Symmetrical expansion: Activation of the central keyhole moves the wedges downward 0.25 mm per turn, similar to a Hyrax expander, resulting in bilateral expansion (Figure 1C).
- Left-sided illustrative asymmetrical expansion: Activation of the left keyhole descends the wedge on that side. The right side, having more structures and surface area, acts as anchorage, allowing primarily left-sided expansion (Figure 1D).
- Right-sided illustrative asymmetrical expansion: Activation of the right keyhole selectively moves the right side, producing conceptual asymmetric expansion (Figure 1E).

### 2.3 Finite element analysis (FEA)

A functional digital simulation of the proposed expander was performed using the finite element method (ANSYS 2024 R1, ANSYS Inc., USA) to assess its mechanical behavior during activation. The device geometry including the center screw, lateral screws, wedges, and expansion arms was imported into ANSYS as a solid model. All components were assigned linear elastic, isotropic material properties corresponding to stainless steel.

**Figure 3:**
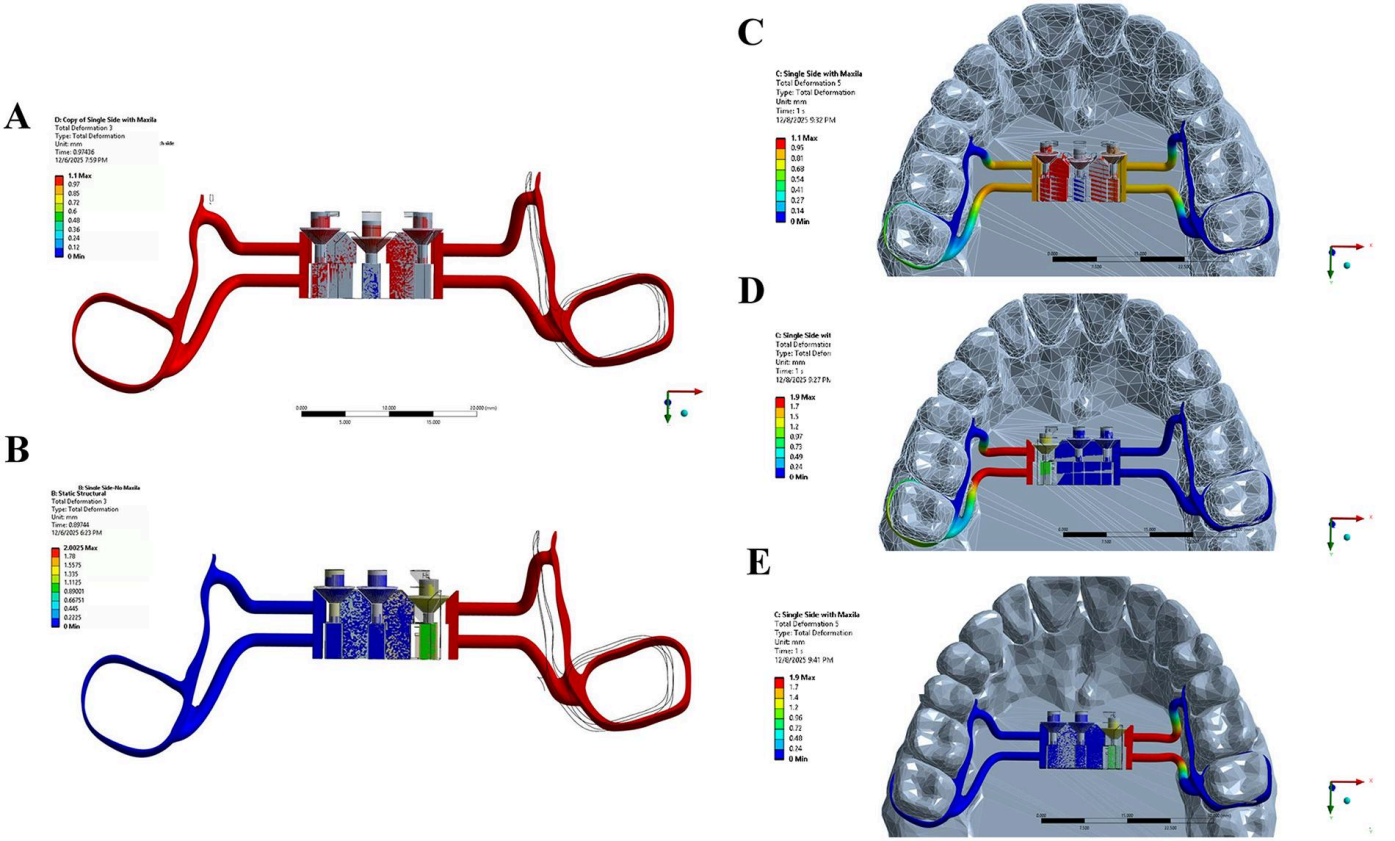
Finite element analysis (FEA) of the innovative electronic key-controlled expander under different activation scenarios. **(A)** Bilateral activation demonstrating symmetric deformation pattern across both sides of the device. **(B)** Single-side activation without maxilla (2 mm deflection) showing increased deformation of the appliance alone. **(C)** Bilateral activation demonstrating symmetric deformation pattern across both sides of the device. **(D)** Left-side activation with maxilla (2 mm deflection) illustrating initial unilateral displacement and stress concentration to the palatal structures. **(E)** Right-side activation with maxilla (2 mm deflection) indicating unilateral displacement and stress distribution transmitted to the palatal structures.

For simulation purposes, the base surface of the device was constrained to represent a simplified anchorage condition, allowing visualization of asymmetric movement. This constraint was applied solely to enable comparative analysis of left- and right-sided activation and does not represent clinical fixation to the palate (Figures 3A–E). Three activation scenarios were applied as controlled displacements:

- 1.0 mm downward movement of the center screw for symmetrical expansion (Figures 3A,C).
- 1.0 mm activation of the left screw with the remaining screws fixed (Figure 3D).
- 1.0 mm activation of the right screw (Figures 3B,E). A tetrahedral mesh with local refinement at screw–wedge interfaces was used.

Outcome variables included lateral displacement, symmetry of movement, and stability of the device under load. The simulation demonstrated smooth bilateral expansion during central activation and isolated unilateral displacement during selective left- or right-side activation.

### 2.4 Device maintenance, sterilization, and safety considerations

The fixed appliance is similar to the conventional Hyrax design and is intended for single use. It is sterilized prior to placement following standard protocols for fixed orthodontic appliances, typically involving ultrasonic cleaning to remove debris, followed by autoclave steam sterilization at 121°C–134°C (11). The electronic key, which externally activates the device, can be reused for multiple patients after high-level disinfection.

Patients are instructed to brush around the expander with a soft toothbrush, use interdental brushes or a water flosser for hard-to-reach areas, and rinse thoroughly after meals.

As the device is currently at the proof-of-concept stage, potential risks include unintended activation, excessive force delivery, or device malfunction. These risks can be mitigated through programmed activation limits, controlled force output, and automatic recording of each activation, including time, date, and applied force. Monitoring and calibration are performed via the electronic key, which connects to a mobile application providing real-time activation tracking and enabling remote clinician oversight.

It is important to emphasize that all proposed advantages remain theoretical at this stage, and both experimental and clinical validation are required before the device can be considered for clinical application.

## 3 Results

The main result of this study is a proof of concept demonstrating the technical feasibility of the electronic key-controlled palatal expander in orthodontic practice. Mechanical behavior was evaluated using FEA, which simulated both symmetric and asymmetric activation. Center-screw activation produced smooth, stable bilateral expansion, with a 1 mm screw advancement resulting in approximately 1.08 mm of lateral displacement (Figures 3A,C and Supplementary Video S1). Selective activation of lateral keyholes yielded illustrative unilateral movement, with each 1 mm screw activation resulting in approximately 1.9 mm of displacement, demonstrating the device's potential for controlled asymmetric expansion under the modeled conditions, with minor contralateral displacement that may be mitigated by anchorage (Figures 3B–E, Supplementary Videos S2, S3). This device might be suitable for integration with AI overlay and could support improvements in dental digital health. The potential advantages of the electronic key-controlled palatal expander compared with conventional and mini-screw-assisted expanders are summarized in Table 1, with Information on the conventional and mini-screw systems drawn from references (12–14).

**Table 1:** Potential advantages of the electronic key-controlled palatal expander compared with conventional and Mini-screw-assisted expanders (proof-of-concept).

Feature	Conventional hyrax expander	Mini-screw-assisted expander	Electronic key-controlled expander (proof-of-concept)
Activation method	Manual screw	Manual screw (hybrid: tooth-borne + bone-borne anchorage with mini-screws)	Electronically guided, programmable
Precision	Symmetric only	Can allow unilateral expansion, but limited	*Designed* for controlled asymmetric activation (not yet validated)
Patient eligibility	Mostly children/adolescents; dependent on caregiver compliance	Adolescents (≥15 years)	Potentially all age groups, including younger children; reduced caregiver dependency
safety	Risk of accidental key swallowing, improper activation	Short-term pain and inflammation, appliance breakage or distortion, potential asymmetrical expansion; major long-term complications rare	Conceptually designed to reduce caregiver errors, accidental key swallowing, and soft-tissue complications; not yet validated
Compliance	Dependent on caregiver	Dependent on caregiver and patient	Potentially patient-friendly, can self-activate, LED guidance to indicate activation site
Monitoring	In-office checks only; no real-time tracking	In-office checks only; limited monitoring	Designed for real-time digital monitoring and remote clinician oversight; includes activation logs (proof-of-concept)
Soft tissue health	Food accumulation under appliance; potential irritation	Food accumulation; gingival inflammation around mini-screws	Convex palatal surface designed to reduce tissue irritation and improve hygiene (proof-of-concept)
Asymmetric expansion	Not controlled; symmetric only	Possible but limited and unpredictable	Designed for programmable, controlled asymmetric expansion (proof-of-concept)

This innovative device has the potential to advance orthodontic care by streamlining the expansion process and enhancing the patient experience. The integration of electronic control and digital monitoring may establish a new standard for managing maxillary transverse deficiencies, offering the possibility of greater precision, efficiency, and improved treatment outcomes.

Beyond its prospective clinical benefits, this technology represents a scalable and investable innovation that could meet the growing demand for efficient, patient-centered orthodontic solutions. Future research should focus on bench and clinical studies to validate its effectiveness and optimize its application.

## 4 Discussion

Previous systematic reviews have primarily examined the effects of orthodontic expanders on dental, hard tissue, and soft tissue changes in the oral cavity (15–17). However, limited attention has been given to their impact on patients' quality of life (18). Orthodontic treatment should not only be evaluated in terms of clinical effectiveness but also in how it affects patients' daily lives and well-being (19).

The present brief research report introduces a novel approach aimed at improving the design of the conventional Hyrax expander, with the potential to positively influence quality of life. Specifically, it presents an electronic key-controlled palatal expander that may be particularly beneficial for both pediatric and adult patients requiring targeted jaw expansion. This device has the potential to deliver precise, automated adjustments, which could enhance treatment safety, improve clinical efficiency, and reduce overall patient's visits.

To better understand the rationale behind this innovation, it is essential to consider the common limitations of the conventional Hyrax expander. The Hyrax is a tooth-borne appliance widely used for rapid maxillary expansion (20). Although several studies have confirmed its effectiveness in producing transverse maxillary expansion and dentoskeletal changes (12, 21, 22), multiple drawbacks have been reported. This include difficulties with screw activation that may reduce patient comfort and compliance (23, 24). Additional concerns involve clinical complications, such as accidental swallowing of the activation key (7), and the reliance on caregivers to perform screw activations accurately (25). Inflammation around the appliance has also been identified as a frequent complication (13). From a biomechanical perspective, the Hyrax primarily permits only symmetric expansion, limiting its use in patients who require asymmetric correction (26). Although some studies have observed unintentional asymmetry with the Hyrax, where one side expands more than the other, this represents a lack of precision rather than a controlled outcome (27). Mini-screw-assisted palatal expanders (MAPE), first introduced by Wilmes et al. (28), provide the possibility of unilateral expansion based on individual needs (29). However, previous studies indicate that MAPE has only been applied in patients aged 15 years and older (30), making it unsuitable for prepubertal children and younger adolescents with unilateral crossbite.

These limitations underscore the need for a more precise, user-friendly, and versatile expansion device. The electronic key-controlled palatal expander described in this brief research has the potential to address many of these shortcomings. By replacing manual activation with electronically guided adjustments, it might reduce caregiver dependency and minimize the risk of complications such as accidental key swallowing. Unlike the Hyrax, its convex palatal surface could improve soft-tissue health and decrease food accumulation beneath the appliance. Additionally, the light LED presence and ease of activation make the patient himself can do the activation. In addition, the design may enhance patient comfort and quality of life by simplifying the activation process and enabling real-time digital monitoring. Most importantly, the inclusion of multiple activation sites introduces the potential for controlled asymmetric expansion under modeled conditions, offering a conceptual alternative for younger patients in whom Mini-screw-assisted devices are not feasible. Although still in the proof-of-concept stage, this device has the potential to offer electronically controlled, programmable activation, broader patient eligibility, and enhanced safety and monitoring features compared with conventional Hyrax and Mini-screw-assisted expanders.

In this brief research report, the primary feature of the electronic key-controlled palatal expander is its ability to perform asymmetric expansion via three independently activated keyholes, allowing adjustments tailored to individual patient needs. A reinforced anchorage is proposed by incorporating additional structural elements on one side, which act as a resistant unit. This anchorage is designed to distribute reactive forces and reduce pressure on the anchor unit (31), ensuring that expansion forces are efficiently directed to the targeted side. Symmetrical expansion can be achieved by activating the central keyhole, which moves the wedges downward by 0.25 mm per turn, similar to a conventional Hyrax expander, resulting in balanced bilateral expansion. Left-sided illustrative asymmetric expansion is achieved by activating the left keyhole; the wedge on that side descends while the right side, with greater structural support, serves as anchorage, allowing primarily left-sided movement. Similarly, right-sided illustrative asymmetric expansion occurs by activating the right keyhole, selectively moving the right side. It is important to note that these biomechanical principles have not yet been tested clinically or experimentally, and this study serves as a proof-of-concept. Future bench testing, refinement, and manufacturing are planned to validate the device's performance and safety.

Although this device is a new concept, our findings are consistent with previous biomechanical studies on maxillary expansion (32–34). Prior finite element analyses of traditional expanders, such as the Hyrax and MARPE, have shown stress concentration along the midpalatal suture and adjacent buttresses, producing combined skeletal and dental effects rather than purely parallel expansion. Most published models use skull-based geometries; however, our simulation focused on the device and maxilla alone, providing a simplified but informative representation of its mechanical behavior. The FEA in this brief report provides preliminary insight into the device's behavior under simulated loading. Central activation produced a uniform stress pattern and symmetrical displacement, while unilateral activation generated illustrative asymmetric deformation localized to the activated side. However, because the palate was fully constrained in our model. As a result, the simulation could not evaluate whether anchorage would truly reduce or eliminate contralateral movement, and the apparent reduction observed under these constraints should be interpreted with caution. Because this analysis used idealized material properties and boundary constraints, the results should be interpreted as proof of concept rather than clinical evidence. Future work incorporating patient-specific anatomy and experimental validation will be essential. Nonetheless, the present FEA offers an initial mechanical foundation for the device, and to our knowledge, electronically driven asymmetric activation has not been previously reported in orthodontic literature.

The clinical relevance of this innovation lies in its potential to address long-standing challenges in palatal expansion, including the need for precise control, asymmetric activation, and reduced reliance on caregiver involvement. By incorporating electronic activation and real-time monitoring, the device may offer a significant advancement over traditional mechanical expanders, particularly for pediatric patients and those requiring unilateral corrections. Furthermore, the integration of teledentistry, defined as delivering dental care services through electronic communication, will enable orthodontists to monitor and manage patients remotely (35). This approach could improve access to care for underserved populations, such as those in rural areas, and ensure continuity of treatment in scenarios like pandemics (35). Looking ahead, the planned incorporation of artificial intelligence and a mobile application into the electronic key system is expected to further enhance functionality, patient engagement, and clinical decision-making.

However, this study has limitations, particularly regarding its technical and clinical applicability. Additional scientific and clinical investigations are necessary before the device can be implemented in practice. A fully functional prototype is planned for development and bench testing to further assess its reliability and feasibility for integration into clinical workflows. Moreover, clinical trials to evaluate its safety, effectiveness, and impact on treatment outcomes in real-world settings are currently in the planning stage. These steps will be essential for determining whether this innovation can be translated into a widely adopted clinical tool.

## 5 Conclusions

The innovative expander may reduce reliance on caregivers, which could improve patient compliance, decrease the likelihood of human error, and enhance overall treatment efficiency. This digitally enhanced device may offer a more user-friendly alternative to conventional Hyrax expanders and has the potential to represent an advancement in orthodontic care. It could be particularly useful for pediatric and special needs patients who require precise, targeted palatal expansion. Additionally, it might offer benefits for patients in rural areas with limited access to hospitals by enabling remote monitoring. However, all proposed advantages remain theoretical, as the device has not yet undergone bench or clinical testing. Experimental and clinical validation will be essential before any clinical application can be recommended. Future studies, including clinical trials, are planned to evaluate its effectiveness and clinical impact.

## Data Availability

The original contributions presented in the study are included in the article/Supplementary Material, further inquiries can be directed to the corresponding author.
