# Supplementary material for: A novel electronic key-controlled expander for precise asymmetric palatal expansion
Source: Front Dent Med. 2026 Jan 12;6:1735298. doi: 10.3389/fdmed.2025.1735298 (PMC12833311; doi:10.3389/fdmed.2025.1735298)
Supplement: Supplementary file 1 [file Supplementaryfile1.docx]

**Supplementary Figures:**

**Supplementary Figure 1.** Traditional Hyrax expander versus innovative expander. **(A)** Traditional Hyrax expander activated manually with a key. **(B)** Innovative expander activated with an electronic key, featuring a unique capability for asymmetric expansion. Concept design of the innovative expander. Three keyholes and a metal arms attached to the teeth. The internal structure illustrates the spring and the slot guiding block movement during activation.

**Supplementary Figure 2.** Outer vertical section of the innovative expander. **(A)** (a) dotted line indicating section plane; (b) keyhole; (c) metal arm transferring force to the jaw. **(B)** Internal view corresponding to section A: (a–c) keyholes, (d) recoil springs, (e) slots permitting block movement after activation, (f) rounded contour of the device preventing food accumulation.

**Supplementary Videos:**

**Supplementary Video 1.** Occlusal-view finite element simulation demonstrating symmetrical expansion following activation of the central keyhole.

**Supplementary Video 2.** Occlusal-view finite element simulation demonstrating left-sided asymmetrical expansion following activation of the left keyhole.

**Supplementary Video 3.** Occlusal-view finite element simulation demonstrating right-sided asymmetrical expansion following activation of the right keyhole.
