# Supplementary figures and images for: A novel electronic key-controlled expander for precise asymmetric palatal expansion
Source: Front Dent Med. 2026 Jan 12;6:1735298. doi: 10.3389/fdmed.2025.1735298 (PMC12833311; doi:10.3389/fdmed.2025.1735298)

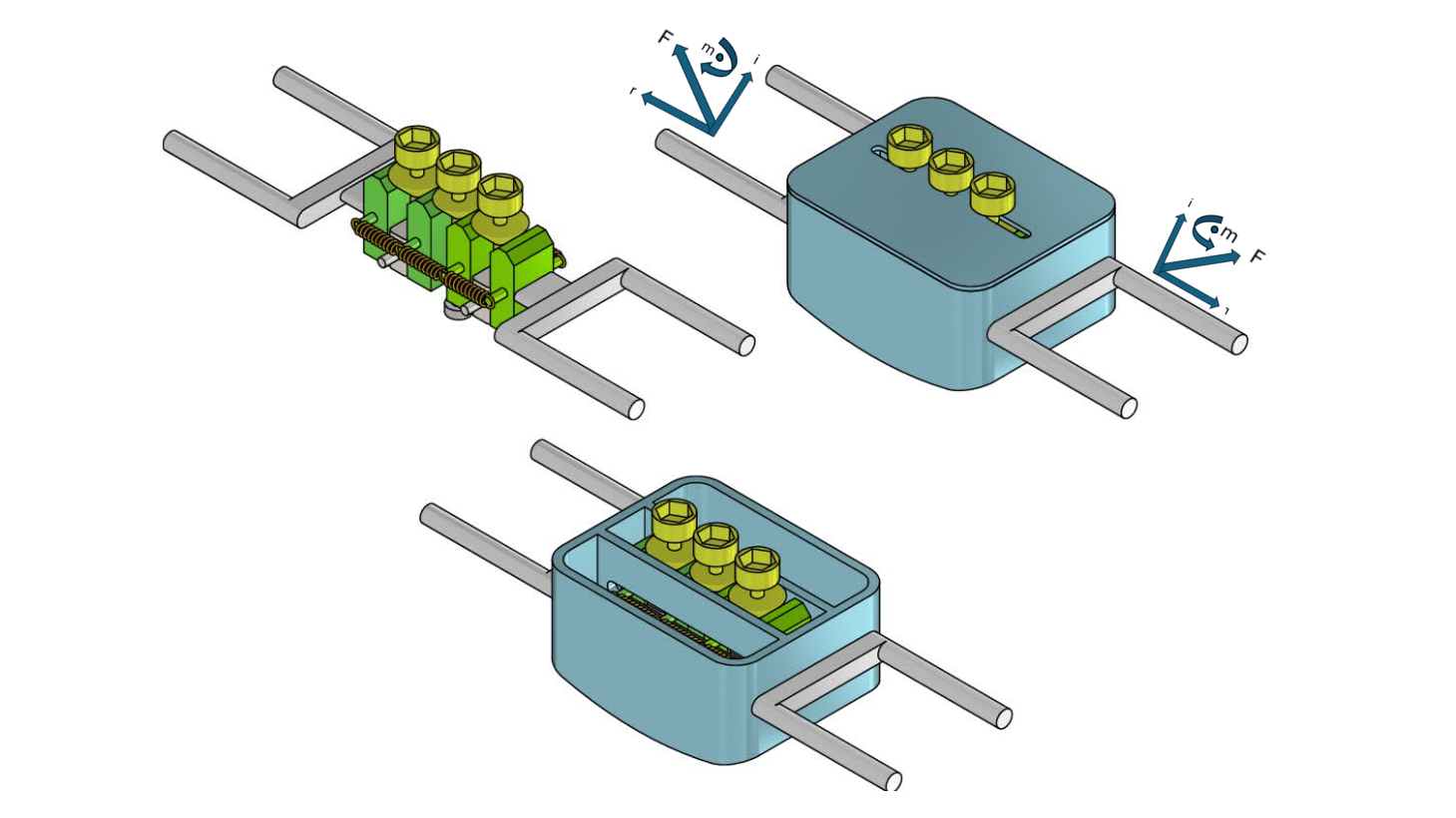

Supplement: Supplementary file 5 [file Image1.tif]

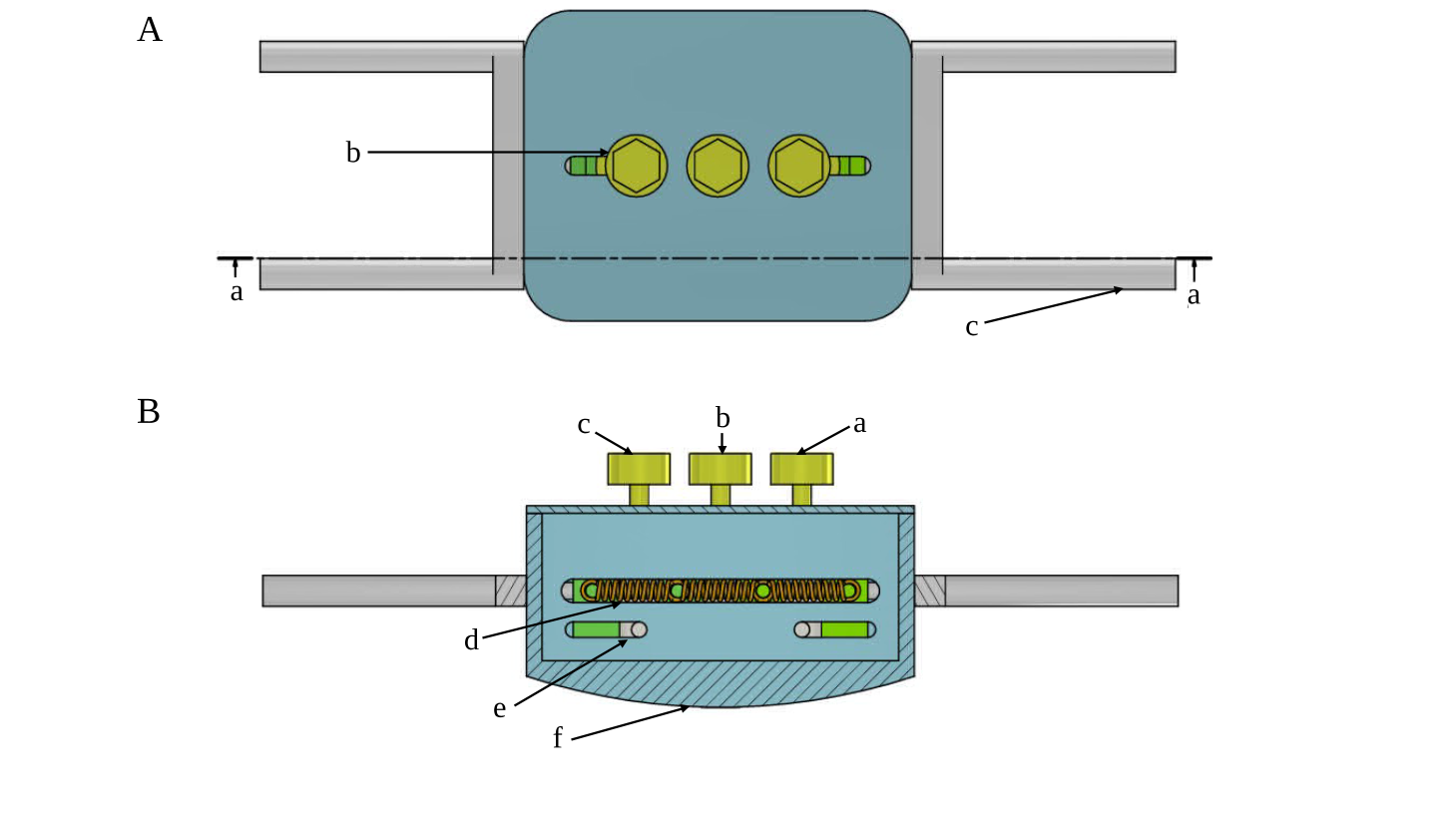

Supplement: Supplementary file 6 [file Image2.tif]
